# Pasteurized Orange Juice Rich in Carotenoids Protects *Caenorhabditis elegans* against Oxidative Stress and *β*-Amyloid Toxicity through Direct and Indirect Mechanisms

**DOI:** 10.1155/2019/5046280

**Published:** 2019-04-18

**Authors:** Ricardo Basílio de Oliveira Caland, Cesar Orlando Muñoz Cadavid, Lourdes Carmona, Leandro Peña, Riva de Paula Oliveira

**Affiliations:** ^1^Rede Nordeste de Biotecnologia (RENORBIO), Universidade Federal do Rio Grande do Norte, Natal, RN, Brazil; ^2^Instituto Federal de Educação, Ciência e Tecnologia do Piauí-IFPI, Brazil; ^3^Fundo de Defesa da Citricultura (Fundecitrus), Araraquara, SP, Brazil; ^4^Instituto de Biología Molecular y Celular de Plantas, Valencia, Spain; ^5^Departamento de Biologia Celular e Genética, Universidade Federal do Rio Grande do Norte, Natal, RN, Brazil

## Abstract

‘Cara Cara' is a red orange (*Citrus sinensis* (L.) Osbeck) variety originally from Venezuela characterized by a significantly higher and diversified carotenoid content including higher-concentration lycopene, *all*-E-*β*-carotene, phytoene, and other carotenoids when compared with the carotenoid profile of its isogenic blond counterpart ‘Bahia', also known as Washington navel. The exceptionally high carotenoid content of ‘Cara Cara' is of special interest due to its neuroprotective potential. Here, we used the nematode *Caenorhabditis elegans* to analyze the antioxidant effect and the protection against *β*-amyloid-induced toxicity of pasteurized orange juice (POJ) obtained from ‘Cara Cara' and compare to that from ‘Bahia'. POJ treatment reduced the endogenous ROS levels and increased the worm's survival rate under normal and oxidative stress conditions. POJ treatment also upregulated the expression of antioxidant (*gcs-1*, *gst-4*, and *sod-3*) and chaperonin (*hsp-16.2*) genes. Remarkably, ROS reduction, gene expression activation, oxidative stress resistance, and longevity extension were significantly increased in the animals treated with ‘Cara Cara' orange juice compared to animals treated with ‘Bahia' orange juice. Furthermore, the body paralysis induced by *β*-amyloid peptide was delayed by both POJs but the mean paralysis time for the worms treated with ‘Cara Cara' orange juice was significantly higher compared to ‘Bahia' orange juice. Our mechanistic studies indicated that POJ-reduced ROS levels are primarily a result of the direct scavenging action of natural compounds available in the orange juice. Moreover, POJ-induced *gst-4::GFP* expression and –increased stress resistance was dependent of the SKN-1/Nrf2 transcription factor. Finally, the transcription factors SKN-1, DAF-16, and HSF-1 were required for the POJ-mediated protective effect against A*β* toxicity. Collectively, these results suggest that orange juice from ‘Cara Cara' induced a stronger response against oxidative stress and *β*-amyloid toxicity compared to orange juice from ‘Bahia' possibly due to its higher carotenoid content.

## 1. Introduction

*Citrus sinensis* L. Osbeck orange juice is considered an excellent dietary source of several bioactive compounds with beneficial properties for human health due to its high content of flavonoids, carotenoids, sugars, minerals, and fiber. Numerous epidemiological and intervention studies have provided substantial evidence to support an inverse correlation between orange juice intake and the occurrence of cardiovascular disease, cancer, and aging-related disorders [1, 2].

Among sweet oranges, there is increasing interest in *C. sinensis* Osbeck cv. ‘Cara Cara', a bud mutation originated from ‘Bahia' navel orange, also known as ‘Washington' navel orange. ‘Cara Cara' orange pulp is characterized by a bright red coloration due to a significantly higher and diversified carotenoid content compared to ‘Bahia' juice, with a mixture of (Z)-isomers of lycopene, *all*-E-*β*-carotene, phytoene, and phytofluene isomers [3]. Total flavanone content as well as hesperidin levels are usually comparable in ‘Cara Cara' and ‘Bahia' pasteurized juice [1]. On the other hand, ‘Bahia' presents a higher ascorbic acid content compared to ‘Cara Cara' [1]. The exceptionally high carotenoid content of ‘Cara Cara' may be of special interest due to recent nutritional studies that have demonstrated its association with the prevention and treatment of various diseases, including neurodegenerative diseases (ND) [4].

Carotenoids are the main pigments responsible for the color of the peel and pulp of citrus fruits. They have been indicated as important dietary nutrients having antioxidant, anti-inflammatory, antimutagenic, anticarcinogenic, and autophagy-modulatory activities [4, 5]. In addition to their direct antioxidant activities, carotenoids can protect cells from oxidative stress by activating the antioxidant network enzymes, including superoxide dismutase (SOD) and catalase (CAT) [4].

Given that oxidative damage and increased neuroinflammation are critically related with the pathogenesis of and neuronal loss in neurodegenerative diseases, the neuroprotective effect of carotenoids has been of specific interest in the search for effective treatments for these diseases. The beneficial effects of dietary carotenoids such as lycopene, astaxanthin, crocin, crocetin, and fucoxanthin on neurodegenerative diseases have been recently studied in animal and cell culture models for Alzheimer's disease (AD) [4]. In a recent paper, Hwang et al. [6] showed that lycopene significantly inhibited intracellular *β*-amyloid accumulation and reduced mitochondrial ROS levels and apoptotic cell death in human neuronal SH-SY5Y cells. In the rodent models, administration of certain types of carotenoids, including lycopene, successfully attenuated not only cellular-level phenotypes such as mitochondrial oxidative damage and increased neuroinflammation but also organism-level phenotypes such as memory impairment and locomotive defects [7]. Despite these studies, there is a substantial lack of AD animal models to show the therapeutic potential of carotenoids against neurodegenerative disease [8–11].

The nematode worm *Caenorhabditis elegans* is an established model organism to study aging and age-related disorders [12–14] and an attractive platform for rapidly screening drug safety and efficacy [15]. It has been demonstrated that lutein supplementation in *C. elegans* is able to suppress the ROS generation induced by the hepatotoxin microcystin-LR (MIC-LR) and restore the levels of the antioxidant enzyme CAT, as well as their survival rate [16]. Pons et al. [17] showed that transgenic *β*-carotene-enriched orange increases *C. elegans* survival under oxidative stress induced by hydrogen peroxide. Furthermore, a delay of paralysis in a *β*-amyloid peptide transgenic *C. elegans* strain was induced by lycopene [18], while fucoxanthin, but not *β*-carotene, provided positive effects on the *C. elegans* lifespan [19].

A number of genes and pathways have been identified to modulate lifespan, stress resistance, and proteostasis in *C. elegans* [20, 21]. These highly conserved pathways include the transcription factors heat shock factor 1 homologue HSF-1, the FOXO homologue DAF-16, and the Nrf-1/2 homologue SKN-1. HSF-1 controls the inducible transcription of a family of genes encoding heat shock proteins (HSPs), many of which are molecular chaperones. The DAF-16/FOXO target genes include specific *hsps* and other stress response/longevity genes. SKN-1 mediates the expression of genes involved in a wide range of detoxification processes, as well as in immunity, proteostasis, and metabolism. Collectively, these transcription factors orchestrate the expression of genes that contribute to longevity.

Up to date, there are no data demonstrating the antioxidant and neuroprotective capacity of pasteurized orange juice (POJ). Here in this work, we assessed and compared the antioxidant capacity and the protective effect against the *β*-amyloid peptide of pasteurized orange juice from ‘Cara Cara' with respect to that from the ‘Bahia' counterpart using the nematode *C. elegans*.

## 2. Materials and Methods

### 2.1. Chemicals, Reagents, and Strains

*tert-*Butyl hydroperoxide (TBHP), fluorodeoxyuridine (FUdR), and 2,7-dichlorodihydrofluorescein diacetate (H_2_DCFDA) were purchased from Sigma-Aldrich (St. Louis, MO, USA).

The following *Caenorhabditis elegans* strains were used: N2 (wild-type strain), EU40 (*skn-1(zu129) IV/nT1 [unc-?(n754) let-?]*) (*IV;V*), CF1038 (*daf-16(mu86*) *I*), PS3551 (*hsf-1(sy441*) *I*), CL2006 (*dvIs2[pCL12(unc-54/human Abeta peptide 1-42 minigene) + pRF4]*), CF1553 (*muIs84 [pAD76(sod-3::GFP)]*), CL2166 (*dvls19[pAF15(gst-4::GFP::NLS)]*), LD1171 (*ldIs3[gcs-1p::GFP + rol-6(su1006)]*), and TJ375 (*gpls[hsp-16.2::GFP]*).

### 2.2. Pasteurized Orange Juice (POJ) Preparation

Pasteurized orange juices obtained from *C. sinensis* L. Osbeck cv. ‘Cara Cara' and cv. ‘Bahia' were provided by Citrosuco (Matão, São Paulo, Brazil). Oranges from both cultivars were collected on June 7th 2016 in Araraquara (São Paulo, Brazil) located in southeastern São Paulo at 23°23′19′′ S and 48°43′22′′ W with an elevation of 600 m above sea level. Orange juices were obtained by cutting and squeezing, using an industrial extractor (model 391, JBT). The pasteurization process was achieved in an industrial pasteurizer (UHT/HTST Lab-25-DH, MicroThermics) at 92− 94°C for 17 s (750 mL/min). After processing, the juices were filled into 1 L plastic flasks and cooled to 1°C. All juices were immediately stored at −20°C.

### 2.3. POJ Characterization

Determination of °Brix/acidity ratio: juice acidity was determined by titration with phenolphthalein and 0.1 N NaOH and was expressed as mg citric acid per 100 g. The soluble solid content (°Brix) was estimated by refractometry, using an Atago® refractometer. The maturity index is expressed as the ratio of °Brix/acidity.

Determination of limonin and vitamin C: limonin extraction was performed by centrifugation of orange juice (50 mL) for 15 min at 5000 rpm. Subsequently, a volume of 2 mL of juice sample was passed through a C18 Sep-Pak cartridge (Waters), and water was added to the concentrated sample and eluted with vacuum using acetonitrile. Finally, the elution was filtered and prepared for HPLC analysis [22]. Vitamin C content was evaluated by the titration method described by Stevens [23].

Carotenoids were extracted as is described in Alquezar et al. [24]. UPLC analysis of individual carotenoids: the carotenoid composition of each sample was analyzed by UPLC with a Nexera X2 Shimadzu liquid chromatography system equipped with a LC-30AD pump and a SPD-M20A photodiode array detector, as well as LabSolutions software (version 5.57 SP1). An Acquity BEH C18 carotenoid column (100 mm × 2.1 mm, 1.8 *μ*m) coupled to a C18 guard column (20 mm × 2.1 mm) (Waters, USA) was used. Samples were prepared for UPLC by dissolving the dried carotenoid extracts in CHCl_3_ : MeOH : acetone (3 : 2 : 1, *v* : *v* : *v*). For carotenoid separation, a binary gradient elution was adapted from the ternary described by Alquezar et al. [24] by using the Gradient Method Calculator (Thermo Scientific). The initial solvent composition consisted of 100% MeOH : water (90 : 5, *v*/*v*) and 5% methyl tert-butyl ether (MTBE). The solvent composition changed in a linear fashion to 95% MeOH : water and 5% MTBE at 0.91 min. During the next minute, the solvent composition was changed to 86% MeOH : water and 14% MTBE. After reaching this concentration, the solvent was gradually changed to 75% MeOH : water and 25% MTBE at 2.28 min, 50% MeOH : water and 50% MTBE 50% at 3.8 min, and 25% MeOH : water and 75% MTBE at 5.32 min. The initial conditions were reached at 6 min, and the column was equilibrated for 2 min before the next injection. The flow rate was 0.5 mL min^−1^, column temperature was set to 27°C, and the injection volume was 3 *μ*L. The photodiode array detector was set to scan from 250 to 800 nm, and for each elution a MaxPlot chromatogram was obtained, which plots each carotenoid peak at its corresponding maximum absorbance wavelength. Carotenoids were identified by their retention time, absorption, and fine spectra [25–29]. The carotenoid peaks were integrated at their individual maxima wavelength, and their contents were calculated using calibration curves of *β*-carotene (Sigma) for *α*- and *β*-carotene; *β*-cryptoxanthin (Extrasynthese) for *β*-cryptoxanthin; zeaxanthin (Extrasynthese) for zeaxanthin; lutein (Sigma) for violaxanthin isomers, mutatoxanthin and antheraxanthin; lycopene (Sigma) for lycopene; and phytoene (Sigma) for phytoene and phytofluene.

### 2.4. *C. elegans* Culture Conditions

For all the experimental procedures, *C. elegans* were cultured at 20°C on Nematode Growth Medium (NGM) plates [30] seeded with *Escherichia coli* OP50. For treatment, worms were cultivated on NGM plates containing 1, 2, 5, or 10% POJ of either ‘Bahia' or ‘Cara Cara'. The control group was prepared in the same manner but without POJ. Tests were also performed with heat-killed *E. coli* OP50 (OP50-HK). Heat-killed bacteria were prepared by incubating liquid cultures of *E. coli* OP50 to 75°C for 1 hour. Synchronized populations were obtained by either bleach treatment or egg-laying.

RNA interference (RNAi) was carried out using the feeding method described previously [31]. Briefly, RNAi clones were grown with 12.5 *μ*g/mL tetracycline and 100 *μ*g/mL ampicillin. On the following day, cultures were diluted in LB supplemented with 60 *μ*g/mL ampicillin and grown to an OD600 of 1. This culture was used to seed plates containing ampicillin and 1 mM IPTG and left to dry for 2 days at room temperature. Synchronized L1 larvae were then placed at 20°C on *E. coli* HT115 that expressed target gene RNAi or control RNAi (empty vector pL4440) for 48 h, until they reached the L4 stage. *skn-1* RNAi efficiency was verified by the absence of F1 larvae. For *daf-16*, RNAi efficiency was confirmed by the suppression of GFP emission on *DAF-16::GFP* transgenic line (TJ356). For *hsf-1*, RNAi efficiency was confirmed by the worms reduced survival at 35°C.

### 2.5. Quantification of Intracellular ROS

N2 wild-type animals synchronized at first-stage larvae (L1) were cultivated on NGM with different concentrations of POJ for 48 h. The experiments were performed under standard and stress conditions as described previously by de Freitas Bonomo et al. [32] with modification. For the stress condition, the animals were exposed to 10 mM *tert*-butyl hydroperoxide (TBHP) in M9 medium for 1 hour. Subsequently, 20 to 40 worms per group were collected in PBS+ 1% Tween-20, washed twice, and transferred to a 96-well microtiter plate, to which 50 *μ*M H_2_DCF-DA was added. Measurements were performed in triplicate in a multilabel microplate reader GloMax®-Multi Detection System (Promega, Wisconsin, USA), with excitation at 490 nm and emission at 510-570 nm, and the mean values were calculated. Readings were performed every 30 min for 4 h.

### 2.6. Reporter Gene Analysis

Transgenic lines expressing *gcs-1::GFP*, *gst-4::GFP*, *sod-3::GFP*, and *hsp-16.2::GFP* were treated with 2% POJ for 48 h at 20°C since L1 until the L4 stage. The experiments were performed under standard and stress conditions. For *gcs-1::GFP*, *gst-4::GFP*, and *sod-3::GFP* animals, the stress condition was induced by incubating in 10 mM TBHP in M9 medium for 1 hour. For *hsp-16.2::GFP*, the stress condition was induced for 1 h at 35°C. Images of 25 worms from each group were acquired using the optic microscope Olympus BX51 (Tokyo, Japan) and fluorescent signals were measured with NIH ImageJ software.

### 2.7. Lifespan and Stress Resistance Assays

The lifespan assay was performed with synchronized N2 wild-type animals treated with 2% POJ starting at the L4 stage. We analyzed approximately 90 animals per group divided into 3 NGM plates containing FUdR to prevent progeny growth. The survival analysis consisted of scoring dead/alive animals every day beginning at the first day of adulthood (*t*0 = day 1) at 25°C. The animals were determined to be dead if no movement was shown with or without stimulation, and those animals with hatched eggs internally, extruded parts, or those who went missing, were excluded from analysis.

To evaluate the resistance to oxidative stress, L4 larval stage of wild-type animals and *skn-1(zu129)*, *daf-16(mu86)*, and *hsf-1(sy441)* mutants were treated with 2% POJ for 48 h on NGM plates containing FUdR seeded with either *E. coli* OP50 (OP50) or heat-killed *E. coli* OP50 (OP50-HK). After that, they were exposed to 10 mM TBHP. Approximately 50 animals were analyzed for each experimental group. Survival fractions were scored every three hours until all animals were considered dead, without pumping or pharyngeal movement. The oxidative stress resistance test was performed three times.

### 2.8. Bioassays for *β*-Amyloid-Induced Paralysis

CL2006 strains, which constitutively express A*β*1–42 peptide in the body wall muscle tissue, were treated since L1 until L4 stage on NGM plates with *E. coli* OP50 for 48 hours at 20°C. Animals were then transferred to new plates containing 2% POJ seeded with either *E. coli* OP50 or OP50-HK for another 48 hours at 20°C. The paralysis phenotype was accelerated by transferring the worms to 35°C. Paralysis was scored at 1 h intervals for up to 12 h. The worms were scored as “paralyzed” based on either the failure of the worms to move their body with the touch of a platinum loop or the formation of a halo on the bacterial lawn indicating a paralyzed condition. Each experiment was performed using at least 30 worms. The data represents mean of three different experiments.

### 2.9. Statistical Analyses

All experiments were performed three times. Statistical analyses were performed using GraphPad Prism (v. 5.0) software (CA, USA). Data were analyzed by Kolmogorov-Smirnov test for normality. For normally distributed data, Student's *t* test was used to compare pairs of groups, whereas a one-way ANOVA followed by Tukey's posttest was used to compare three or more groups. Nonparametric data were analyzed using the Mann–Whitney test when comparing two groups and the Kruskal-Wallis test followed by Dunn's posttest for comparing three or more groups. Survival curves were analyzed by the log-rank (Mantel-Cox) test. For all tests, statistical significance was determined as *p* < 0.05.

## 3. Results and Discussion

### 3.1. Quality and Phytochemical Characterization of Pasteurized Juice from ‘Cara Cara' and ‘Bahia' Oranges

After pasteurization, we compared some quality and phytochemical compounds of the ‘Bahia' sweet orange juice and its spontaneous red-fleshed mutant ‘Cara Cara', which contains large proportions of linear carotenes. Pasteurized ‘Bahia' juice (PBJ) was more acidic than pasteurized ‘Cara Cara' juice (PCJ) (Table 1). A higher content of ascorbic acid and limonin was recorded in PBJ compared to PCJ (Table 1). As expected, the total carotenoid content was significantly higher in ‘Cara Cara' juice compared to ‘Bahia', especially *Z*-violaxanthin, zeaxanthin, phytoene, phytofluene, lycopene, and *β*-carotene (Table 2). These results are comparable essentially to those of the previous studies by Brasili et al. [1] and Lee [3], which showed that lycopene and *β*-carotene accumulated at high levels in PCJ but their concentrations were very low or undetectable in PBJ.

### 3.2. POJ Treatment Reduces Intracellular ROS Accumulation Primarily as a Result of Direct Scavenging

**Figure 1:**
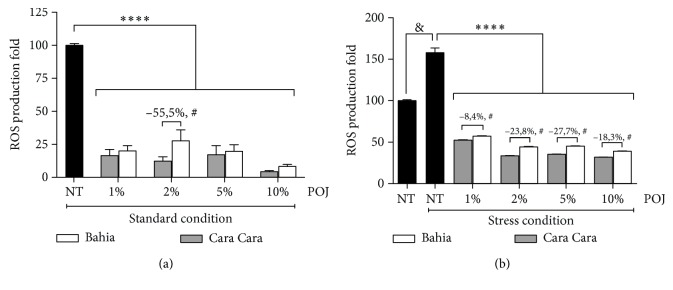
Effect of pasteurized juice from ‘Bahia' and ‘Cara Cara' oranges on intracellular ROS accumulation in *C. elegans*. (a) Wild-type animals were treated with either 1, 2, 5, or 10% of ‘Bahia' or ‘Cara Cara' juice for approximately 48 h since L1. ROS production was measured using the dye H_2_DCFDA. Results are expressed as H_2_DCFDA fluorescence levels. ^∗∗∗∗^*p* < 0.0001 compared to respective not treated (NT) control and ^#^*p* = 0.0026 comparing 2% ‘Bahia' with 2% ‘Cara Cara' juice by 2-way ANOVA. (b) Wild-type animals were treated with either 1, 2, 5, or 10% of ‘Bahia' or ‘Cara Cara' juices for approximately 48 h since L1 and then incubated on 10 mM TBHP for 1 hour to induce oxidative stress. Results are expressed as mean H_2_DCFDA fluorescence levels ± SEM of values. ^&^*p* < 0.0001 compared to NT under standard conditions by a two-tailed Student's *t*-test, ^∗∗∗∗^*p* < 0.0001 compared to respective NT control under stress and ^#^*p* < 0.0001 comparing ‘Bahia' orange with ‘Cara Cara' juice in each concentration by 2-way ANOVA. Percentage difference (%) between ‘Bahia' and ‘Cara Cara' juices are indicated for those with ^#^statistically significance.

In order to determine the antioxidant capacity of POJ from ‘Bahia' and ‘Cara Cara' orange, we investigated the influence of the POJ treatment on the intracellular accumulation of ROS in *C. elegans*. Under standard condition, wild-type worms treated with 1, 2, 5, and 10% of either ‘Bahia' or ‘Cara Cara' juice displayed reduced ROS levels compared to the control group of untreated worms (*p* < 0.0001) (Figure 1(a)). Interestingly, ROS levels were significantly lower in the animals treated with 2% ‘Cara Cara' juice compared to those from worms treated with 2% ‘Bahia' juice (*p* = 0.0026) (Figure 1(a)). Similar results were also observed when the worms were treated with POJ and then subjected to stress conditions (Figure 1(b)). All concentrations tested of POJ were able to reduce ROS production under stress conditions to a level below to those observed in the animals subjected to standard condition (*p* < 0.0001) (Figure 1(b)). Under stress conditions, ‘Cara Cara' juice reduced ROS production more efficiently than ‘Bahia' juice did in all concentrations tested (*p* < 0.0001). Since 2% ‘Cara Cara' juice was the only concentration that showed better results than ‘Bahia' juice under standard conditions, we performed all following assays using 2% POJ treatment.

Phytochemicals have the potential to modulate intracellular oxidative stress directly by scavenging free radicals. Besides that, they are also able to modulate oxidative stress indirectly by upregulating antioxidant and phase II detoxification enzymes which are the major enzymatic line of defense against electrophilic toxicants and oxidative stress. The induction of these adaptive systems by phytochemicals may be related to the fact that they function as xenobiotics in animals [33, 34]. The metabolism of xenobiotics/phytochemicals can produce reactive species, reactive intermediates, and metabolites that can act as prooxidants [35]. Therefore, phytonutrients would play a role as a mild stress trigger leading to the activation of defense mechanisms for their own detoxification, which in turn could induce organisms' resistance to a more severe oxidative stress condition. In *C. elegans*, these defense mechanisms are at least partly a result of the activation of three transcription factors, DAF-16, SKN-1, and HSF-1 [36].

**Figure 2:**
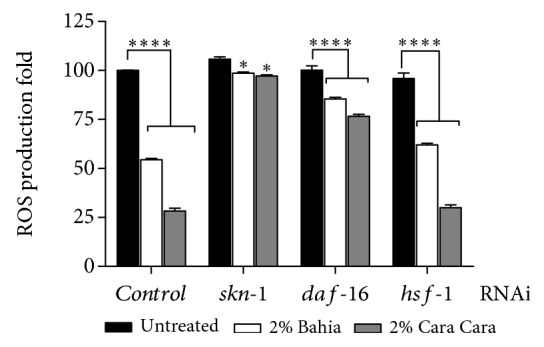
Contribution of SKN-1, DAF-16, and HSF-1 on ROS accumulation in POJ-treated worms. RNAi animals were treated with 2% POJ for approximately 48 h since L1. ROS production was measured using the dye H_2_DCFDA. Results are expressed as mean H_2_DCFDA fluorescence levels ± SEM of values. ^∗∗∗∗^*p* < 0.0001, ^∗∗^*p* < 0.0016, and ^∗^*p* < 0.0111 compared to respective untreated RNAi by 2-way ANOVA.

In order to elucidate whether ROS reduction was related to POJ direct or an indirect mechanism of action, we performed ROS quantification in animals submitted to RNAi for the SKN-1, DAF-16, and HSF-1 transcription factors. Under standard conditions, knockdown of *skn-1*, *daf-16*, and *hsf-1* significantly reduced the ROS production in the worms treated with 2% of either ‘Bahia' or ‘Cara Cara' juices (Figure 2) suggesting that ROS reduction induced by POJ may be independent of these transcription factors. Interestingly, quantification of ROS levels showed that the lowest reduction was on *skn-1(RNAi)* animals (Figure 2). The ROS level diminished only by 6.75 and 8.18% on *skn-1(RNAi)* worms treated with 2% ‘Bahia' and 2% ‘Cara Cara' juices, respectively. Meanwhile, ROS reduction was 14.48% and 23.35% on *daf-16(RNAi)* animals and 33.88% and 66% on *hsf-1(RNAi)* animals treated with 2% ‘Bahia' or ‘Cara Cara' juices, respectively. This result suggests that ROS reduction promoted by POJ treatment was primarily a result of the direct scavenging action of the carotenoids and other phytonutrients present in the orange juice and secondly an outcome of the transcription factor SKN-1 indirect action.

### 3.3. POJ Treatment Induces the Expression of Antioxidant, Detoxification, and Chaperonin Genes

**Figure 3:**
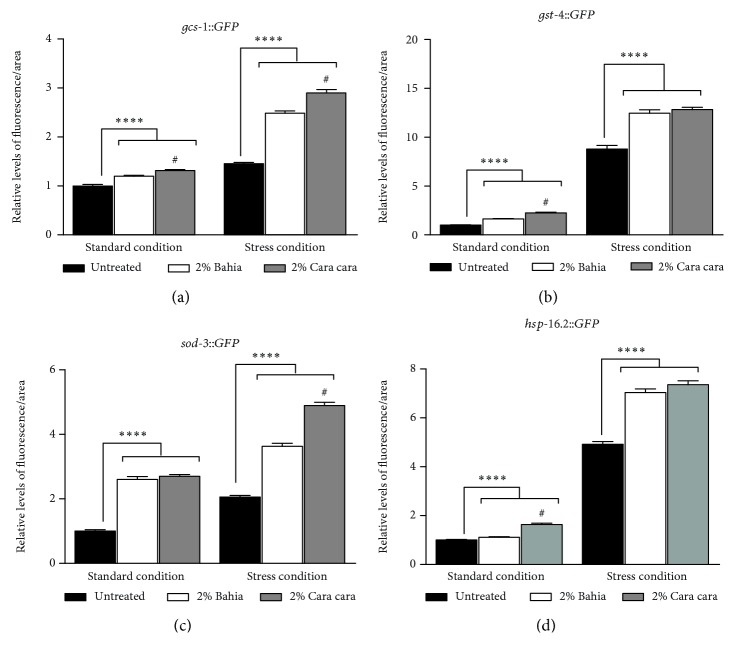
Effect of pasteurized juice from ‘Bahia' and ‘Cara Cara' oranges on the expression of antioxidant and detoxification genes. Analysis of *gcs-1::GFP* (a), *gst-4::GFP* (b), *sod-3::GFP* (c), and *hsp-16.2::GFP* (d) fluorescent expression levels. Transgenic worms were treated or not with 2% ‘Bahia' or 2% ‘Cara Cara' juice for 48 h. After this period, animals were submitted to stress conditions. For *gcs-1::GFP* (a), *gst-4::GFP* (b), and *sod-3::GFP* (c); the stress condition was incubation for 1 h on 10 mM TBHP. For *hsp-16.2::GFP*, the stress condition was incubation for 1 h at 35°C. Photographs were taken on a fluorescence microscope, and GFP fluorescence signals were measured using NIH ImageJ software. The results represent mean GFP levels ± SEM of values. ^∗∗∗∗^*p* < 0.0008 by a two-tailed Student's *t-*test compared to the untreated group, ^#^*p* < 0.0003 by a two-tailed Student's *t-*test for 2% ‘Bahia' with 2% ‘Cara Cara' juice.

To better characterize the molecular responses associated with the direct and indirect antioxidant effects of POJ in *C. elegans*, we analyzed the gene expression of four reporter genes related to stress resistance, detoxification, and longevity. We selected *γ*-glutamyl cysteine synthetase (*gcs-1*) and glutathione-s-transferase-4 (*gst-4*), two SKN-1 target genes [37, 38], superoxide dismutase 3 (*sod-3*), a well-known DAF-16 target gene [39], and the chaperone *hsp-16.2::GFP* whose expression is regulated by DAF-16 and HSF-1 [40]. The fluorescence signals of *gcs-1::GFP*, *gst-4::GFP*, *sod-3::GFP*, and *hsp-16.2::GFP* animals were significantly increased after 2% POJ treatment with either ‘Bahia' or ‘Cara Cara' juices compared to untreated worms, both at standard and stress conditions (Figures 3(a)–3(d)). Interestingly, the expression signal of *gcs-1::GFP* (Figure 3(a)), *gst-4::GFP* (Figure 3(b)), and *hsf-16.2::GFP* (Figure 3(d)) from the animals treated with 2% ‘Cara Cara' juice were significantly higher than that of the animals treated with 2% ‘Bahia' juice under standard conditions. Under stress conditions, the treatment with 2% ‘Cara Cara' juice significantly increased the expression of *gcs-1::GFP* and *sod-3::GFP.*

The POJ induction, rather than an inhibition, of the antioxidant and detoxification genes indicates that the phytonutrients present in the POJ were not only directly scavenging ROS but also acting as prooxidants and inducing a mild cellular stress response [41, 42]. This phenomenon is also referred as hormesis since the phytochemicals are toxic and protect plants against insects and other harmful organisms and stresses. However, at the subtoxic doses, the phytochemicals involve kinases and transcription factors in order to induce the expression of genes that encode antioxidant enzymes, protein chaperones, phase-2 enzymes, neurotrophic factors, and other cytoprotective proteins [41, 42].

**Figure 4:**
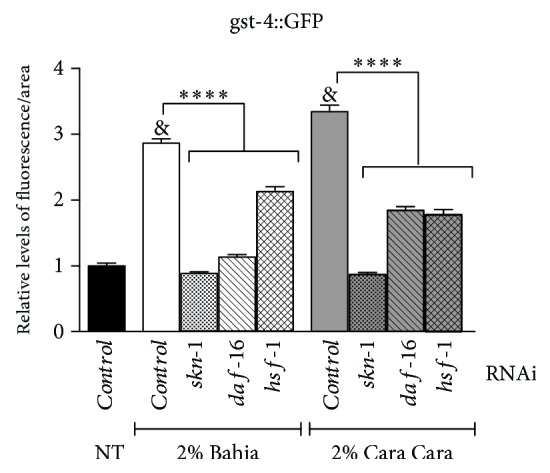
Contribution of SKN-1, DAF-16, and HSF-1 on *gst-4::GFP* expression induced by POJ treatment. RNAi animals were treated with 2% POJ for approximately 48 h since L1. Photographs were taken on a fluorescence microscope, and GFP fluorescence signals were measured using NIH ImageJ software. The results represent mean GFP levels ± SEM of values. ^&^*p* < 0.0001 compared to untreated (NT) *control(RNAi*) under standard conditions and ^∗∗∗∗^*p* < 0.0001, compared to respective POJ-treated *control(RNAi)* by 1-way ANOVA.

Since *gst-4::GFP* showed the strongest expression after POJ treatments, we assessed whether it was dependent on SKN-1, DAF-16, and HSF-1. RNAi of all three transcription factors reduced significantly *gst-4::GFP* expression induced by the POJ treatments suggesting that they all contribute to its activation (Figure 4). Moreover, knocking down of *skn-1* caused the greatest reduction on *gst-4::GFP* expression induced by the POJ treatments. The fluorescent levels of *gst-4::GFP* in the worms treated with ‘Bahia' juice were 3.26 times lower on *skn-1(RNAi)* animals compared to *control(RNAi)* animals while the reduction was 2.54 and 1.34 times lower on *daf-16(RNAi)* and *hsf-1(RNAi)* animals, respectively. This result suggests that SKN-1 seemed to play a more important role on *gst-4* expression compared to DAF-16 or HSF-1.

### 3.4. POJ Treatment Increases Longevity and Oxidative Stress Resistance

**Figure 5:**
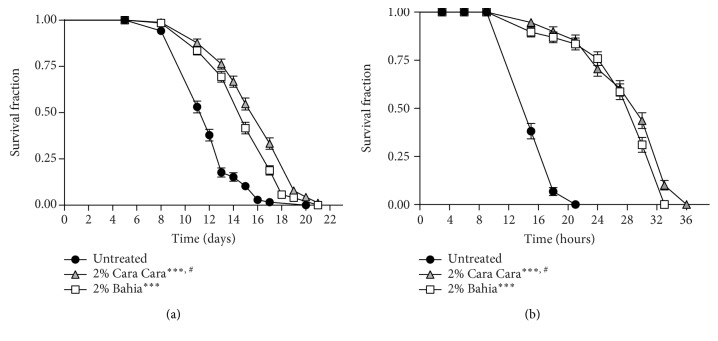
Effect of pasteurized juice from ‘Bahia' and ‘Cara Cara' oranges on *C. elegans* stress resistance and longevity. (a) Survival curves of wild-type (N2) animals under standard laboratory conditions. Worms were treated with either 2% ‘Bahia' or 2% ‘Cara Cara' juice beginning at L4. Survival was verified every day at 25°C. ^∗∗∗∗^*p* < 0.0001 compared to respective controls and ^#^*p* = 0.0003 comparing animals treated with 2% ‘Bahia' with 2% ‘Cara Cara' juice by log-rank (Mantel-Cox) test. (b) Survival curves of wild-type (N2) animals under oxidative stress conditions. Worms were treated with either 2% ‘Bahia' or 2% ‘Cara Cara' juice beginning at L4 and transferred to plates with 10 mM TBPH. Survival was verified every 3 hours at 20°C. ^∗∗∗∗^*p* < 0.0001 compared to the untreated control group and ^#^*p* = 0.0134 comparing animals treated with 2% ‘Bahia' with 2% ‘Cara Cara' juice by log-rank (Mantel-Cox) test.

To explore whether POJ antioxidant properties have a protective effect *in vivo*, we tested whether POJ could affect the lifespan of *C. elegans* under standard laboratory conditions. We determined the lifespan of N2 worms with and without 2% POJ treatment. The maximum and mean lifespan of N2 worms fed with either 2% pasteurized ‘Bahia' or ‘Cara Cara' juice was significantly increased compared to untreated worms (Figure 5(a), Table 3). Remarkably, animals treated with 2% ‘Cara Cara' juice showed an increased lifespan compared to animals treated with 2% ‘Bahia' juice (*p* = 0.0003) (Table 3).

Several studies have shown that lifespan extension is closely associated with enhanced resistance to various forms of environmental stressors [43]. Thus, we assessed the effects of the POJ treatments on *C. elegans* oxidative stress resistance. Oxidative stress assays were performed in wild-type animals treated with 2% POJ for 48 h and then submitted to stress conditions induced by *tert-*butyl hydroperoxide (TBHP). We observed that animals treated with either 2% ‘Bahia' or ‘Cara Cara' juice showed an increased maximum and mean lifespan, when compared to untreated controls (Figure 5(b), Table 4). Similar to the longevity assay, animals treated with 2% ‘Cara Cara' juice showed increased oxidative stress resistance compared to animals treated with 2% ‘Bahia' juice (*p* = 0.0134).

We also repeated the oxidative stress resistance assay in *skn-1*, *daf-16*, and *hsf-1* knockout animals. POJ treatment with either 2% ‘Bahia' or ‘Cara Cara' juices significantly increased the maximum and mean survival time of *daf-16* and *hsf-1* mutants under stress conditions (Table 4). However, treatment with either 2% POJ ‘Bahia' or ‘Cara Cara' failed to increase *skn-1* mutant survival under stress conditions (Table 4). These results suggest that the oxidative stress resistance induced by POJ could be mediated by the transcription factor SKN-1. In this work, POJ antioxidant capacity was positively related to an increase in survival under standard and oxidative stress conditions which would be in agreement with the free radical theory of aging.

Together, these results indicate that pasteurized juice treatment improves *C. elegans* antioxidant capacity, which is associated with the upregulation of antioxidant and chaperonin genes and increased lifespan and stress resistance. It is noteworthy that the treatment with ‘Cara Cara' orange juice provided better results on all parameters analyzed here compared to ‘Bahia' orange juice with the only exception for the expression of *sod-3::GFP* (Figure 3(c)) under standard conditions and for *gst-4::GFP* (Figure 3(b)) and *hsp-16.2* (Figure 3(d)) under stress conditions. The beneficial effects of ‘Cara Cara' orange juice could be related to its higher carotenoid content, especially *Z*-violaxanthin, phytoene, phytofluene, lycopene, and *β*-carotene, even though ‘Bahia' juice has higher levels of vitamin C and limonin (Table 2). However, the additional effects of ‘Cara Cara' orange juice compared to ‘Bahia' orange juice are either moderate or small to be explained solely based on the total carotenoid difference between these two varieties.

### 3.5. POJ Treatment Delays Paralysis Induced by *β*-Amyloid (A*β*) Expression

**Figure 6:**
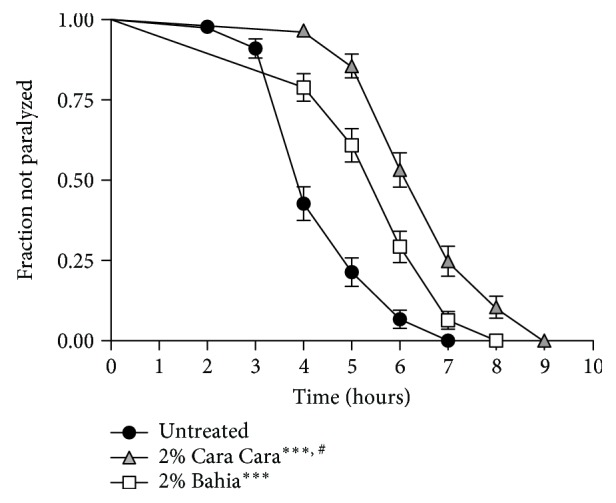
Effect of pasteurized juice from oranges cv. ‘Bahia' and cv. ‘Cara Cara' treatment on the *β*-amyloid induced paralysis in *C. elegans* transgenic model of Alzheimer's disease. Paralysis curves for *C. elegans* strain CL2006 which expresses *β*-amyloid peptide constitutively in the muscle. Worms were treated beginning at L4 with 2% POJ for 48 h at L4 stage. Paralysis was verified at 1 h intervals at 35°C. ^∗∗∗∗^*p* < 0.0001 compared to untreated control and ^#^*p* < 0.0001 comparing 2% ‘Bahia' with 2% ‘Cara Cara' juice by log-rank (Mantel-Cox) test.

The oxidative stress state has an important role in the development of neurodegenerative diseases such as Alzheimer's disease (AD). Accumulation of ROS and deposition of toxic amyloid species have been proposed to exacerbate the symptoms observed in AD patients [44, 45]. The observation that POJ has antioxidant properties *in vivo* led us to ask whether POJ could protect against *β*-amyloid-induced toxicity in a *C. elegans* model for Alzheimer's disease. The expression of human A*β*1–42 in the muscle of transgenic CL2006 strain promotes a paralysis that can be monitored over time. We observed that the onset of paralysis was significantly delayed in POJ-treated animals (Figure 6). The mean paralysis time for worms treated with 2% ‘Bahia' and 2% ‘Cara Cara' juices was increased by 25.0% and 46.0%, respectively, compared to untreated worms (Figure 6, Table 5).

Various studies have shown that the protective effect of flavonoids and carotenoids against amyloid-induced neurotoxicity is due to their antioxidant properties. Other possible mechanisms could be attenuation of A*β* aggregation *in vivo* by downregulating the expression level of *β*-amyloid precursor protein (APP) [18] and/or activation of protein degradation pathways [46]. In the present study, the POJ treatment delayed A*β*1–42-induced paralysis in worms suggesting that POJ may be able to attenuate the development of AD by altering A*β* aggregate states *in vivo*. Most importantly, our results indicate that ‘Cara Cara' juice, with higher carotenoid contents, provides a superior protection against A*β*1–42-induced paralysis over that provided by Bahia.

We also evaluated the role of SKN-1, DAF-16, and HSF-1 in POJ-mediated protection against A*β*1-42 toxicity. To this end, we examined the effect of POJ on CL2006 worms using RNAi to knock down *skn-1*, *daf-16*, and *hsf-1* expression. POJ treatment significantly delayed the paralysis rate of CL2006 worms with *control(RNAi)* (Table 5). In contrast, RNAi of *skn-1* and *daf-16* in CL2006 worms completely abolished POJ-mediated beneficial effects on delaying the progression of paralysis (Table 5). RNAi of *hsf-1* in CL2006 worms only prevented the beneficial effect against *β*-amyloid paralysis for those animals treated with 2% ‘Cara Cara' juice (Table 5). Together, these findings suggest that the transcription factors SKN-1, DAF-16, and HSF-1 are required for POJ-mediated protective effect against A*β* toxicity.

Taking these data together, our mechanistic studies indicated that POJ improves the antioxidant status of a whole organism by direct and indirect mechanisms. POJ-reduced ROS levels were primarily a result of the direct scavenging action of natural compounds available in the orange juice and secondly an outcome of the transcription factor SKN-1 indirect action. POJ promotes *gst-4::GFP* expression and oxidative stress resistance mainly through SKN-1 although DAF-16 and HSF-1 also contribute to a less extent to these effects. Finally, POJ delayed A*β*-induced onset paralysis on a SKN-1-, DAF-16-, and HSF-1-dependent manner. Previous studies in the nematode reported that DAF-16, SKN-1, and HSF-1 play pivotal roles in regulating longevity and ameliorating A*β* [12]. Since these transcription factors are key regulators of many important biological processes, including lifespan, stress responses, and proteostasis, we reasoned that POJ treatments might protect worms against A*β*1–42 toxicity by increasing antioxidant capacity and proteostasis.

### 3.6. POJ Protection against Oxidative Stress and A*β* Toxicity Is Partially Related to Antimicrobial Effect

**Figure 7:**
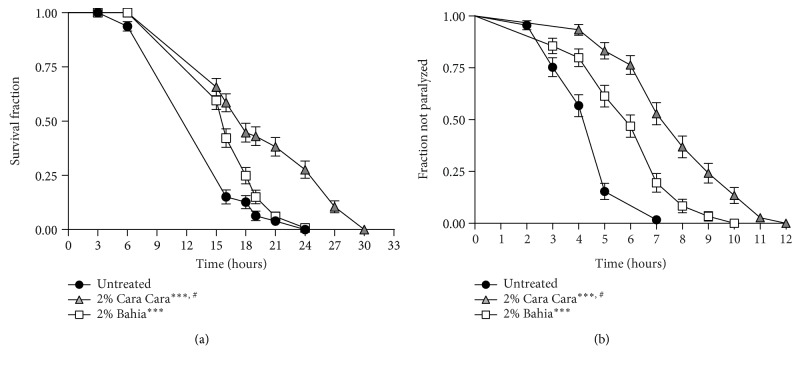
Effect of POJ on oxidative stress resistance and A*β*1-42-induced paralysis on *C. elegans* grown on dead bacteria. (a) Stress resistance assay on bacteria dead bacteria. Worms were treated with 2% POJ on *E. coli* OP50 heat-killed beginning at L4 and transferred to plates with 10 mM TBPH. Survival was verified every 3 hours at 20°C. ^∗∗∗∗^*p* < 0.0001 compared to the untreated control group, log-rank (Mantel-Cox) test. (b) Paralysis profile of CL2006 transgenic animals fed with 2% POJ mixed with OP50 heat-killed. Paralysis was verified at 1 h intervals at 35°C. ^∗∗∗∗^*p* < 0.0001 compared to untreated control by log-rank (Mantel-Cox) test.

Since *E. coli* has a pathogenic effect on *C. elegans*, which may alter its longevity, resistance to stress, and A*β*1–42-induced paralysis [47, 48], we investigated whether the POJ protective effects could be a secondary response of a possible POJ antimicrobial property. First, we repeated the oxidative stress resistance assay in animals treated with POJ on dead bacteria. We observed that wild-type animals treated with 2% POJ still showed increased survival on 10 mM TBHP compared to untreated controls (*p* < 0.0001) (Figure 7(a), Table 4). However, the mean survival variation observed for the POJ-treated animals on dead bacteria was considerably lower compared to the, respectively, POJ-treated animals on live bacteria (Table 4). The mean survival variation was 11 and 68% for the animals treated with 2% ‘Bahia' juice on dead and live bacteria, respectively. Likewise, the mean survival variation was 32 and 73.5% for the animals treated with 2% ‘Cara Cara' juice on dead and living bacteria, respectively (Table 4).

The role of bacterial in affecting longevity of aging *C. elegans* is well known [49]. Much experimental evidence supports bacterial infection and proliferation within the intestine of adults as a potential pathogen that triggers innate immune and stress host responses which may contribute as a double-edged effect to tissue damage and aging [47]. The conserved PMK1-p38 mitogen-activated protein kinase (MAPK) pathway is the major mediator of innate immunity in *C. elegans*. Interestingly, the PMK-1 pathway is also required for the activation of the transcription factor SKN-1 under stress conditions [50]. Thus, any intervention that attenuates bacterial pathogenicity and toxicity extends lifespan and stress resistance. The reduction of the variation survival time on stress when the POJ-treated animals were fed on bacterial previously killed by heat indicates that part of the survival extension observed on POJ-treated animals fed on live bacteria is due to its antimicrobial effect. It is also reasonable to suppose that this reduction of the variation survival time on stress could be a result of the animals acquiring less antioxidant buffer from their living bacterial food source [49]. Another possibility could be related to the fact that in the absence of pathogenic bacteria, the PMK-1-SKN-1 signaling pathway is also diminished and therefore the defense mechanisms activation is attenuated.

Next, we performed the A*β*1-42-induced paralysis assay also using bacteria killed by heat. Interestingly, the mean variation observed for the A*β*1-42-induced paralysis was not particularly different between the POJ treatments on dead or living bacteria (Figure 7(b), Table 5). The mean paralysis time variation for the animals treated with 2% ‘Bahia' juice on dead bacteria was 23% compared to untreated animals while the variation for the same treatment on living bacteria was 25% (Table 5). For ‘Cara Cara' juice treatment, the mean paralysis time variation was 48% compared to untreated animals on dead bacteria and 46% compared to untreated animals on living bacteria (Table 5).

Although the onset paralysis in the CL2006 animals grown on live OP50 was faster compared to those grown on dead OP50 as expected, we did not observe any significant difference of the paralysis time variation on the POJ-treated animals fed on dead bacteria compared to those fed on live bacteria (Table 5). This result suggests that the beneficial effect of POJ against A*β* toxicity is independent of the bacterial pathogenicity. This observation seems contradictory since many studies have shown a link between food source and proteotoxicity [48, 51]. Steinkraus et al. [51] showed that bacterial food deprivation suppresses proteotoxicity in A*β* worms through an *hsf-1*-dependent mechanism. It would be interesting for further studies to shed light on the possible relationship between POJ protection against A*β* in the absence of bacteria depends on HSF-1.

## 4. Conclusions

In this work, we investigated the *in vivo* effects of POJ from the ‘Bahia' and ‘Cara Cara' varieties using the *C. elegans* model. Treatment with POJ reduced the endogenous levels of ROS and increased the rate of survival of the worms under normal and stress conditions. Our mechanistic studies indicated that POJ promotes resistance to oxidative stress by acting through the transcription factor SKN-1. We observed that POJ treatments increased the expression of the antioxidant genes (*gcs-1*, *gst-4*, and *sod-3*) and chaperonin (*hsp-16.2*), which are known to be regulated by the transcription of SKN-1, DAF-16, and HSF-1 factors. In addition, POJ treatments delayed *β*-amyloid-induced paralysis in the transgenic model *C. elegans* in a manner requiring SKN-1, DAF-16, and HSF-1. Noteworthy, the treatment with the two juice types produced excellent results; however, ‘Cara Cara' juice induced significantly better responses than ‘Bahia' juice did in almost all experiments, possibly due to its higher content of carotenoids such as *Z*-violaxanthin, zeaxanthin, phytoene, phytofluene, lycopene, and *β*-carotene.

## Figures and Tables

**Table 1:** Quality parameters of pasteurized juices from ‘Bahia' and ‘Cara Cara' oranges.

Parameters	Bahia	Cara Cara
°Brix	10.8 ± 0.2	9.5 ± 0.1
Acidity (g/100 g)	0.8 ± 0.0	0.5 ± 0.0
Ratio °Brix/acidity	14.1 ± 0.3	18.6 ± 0.1
Vitamin C (mg/L)	424.9 ± 20.7	215.7 ± 9.8
Limonin (mg/L)	7.4 ± 1.4	2.7 ± 0.3

**Table 2:** Content of carotenoids in pasteurized juices from ‘Bahia' and ‘Cara Cara' oranges.

Carotenoid (ng/mL)	Bahia	Cara Cara
*E*-violaxanthin	Tr.	Tr.
*Z*-Luteoxanthin^a^	34.52 ± 5.26	n.d.
*all*-*E*-Luteoxanthin^a^	271.89 ± 32.79	n.d.
*Z*-Violaxanthin	815.68 ± 26.42	1021.94 ± 109.94
Zeaxanthin^a^	Tr.	195.01 ± 48.78
Anteraxanthin	20.82 ± 8.27	n.d.
Mutatoxanthin^a^	66.09 ± 9.92	n.d.
Zeinoxanthin^a^	196.31 ± 10.6	n.d.
*β*-Cryptoxanthin	456.50 ± 14.08	351.07 ± 4.69
Phytoene	n.d.	6172.03 ± 113.78
Phytofluene	n.d.	2661.61 ± 136.84
Lycopene	n.d.	469.05 ± 26.55
*α*-Carotene^a^	n.d	Tr.
*β*-Carotene	n.d.	228.89 ± 78.47
Total^b^:	1564.81 ± 70.41	11099.25 ± 158.88

n.d.: not detected. Tr.: traces. ^a^Identified tentatively, ^d^total carotenoids calculated as the sum of all the carotenoids identified individually.

**Table 3:** Effect of pasteurized juice from ‘Bahia' and ‘Cara Cara' oranges on *C. elegans* lifespan.

Condition	Maximum survival (days ± SEM)	Mean survival (days ± SEM)	% mean survival time variation vs. untreated	*p* value (log rank) POJ^a^ vs. untreated^b^	*p* value (log rank) PBJ^c^ vs. PCJ^b,d^	*N* ^e^
Untreated	17.33 ± 2.31	12.11 ± 0.13				243 (3)
2% Bahia	19.00 ± 1.73	14.75 ± 0.17	22.0	<0.0001		255 (3)
2% Cara Cara	19.33 ± 2.31	15.64 ± 0.18	29.5	<0.0001	0.0003	234 (3)

^a^POJ, pasteurized orange juice, ^b^comparisons were performed using log-rank (Mantel-Cox) test. ^c^PBJ, pasteurized ‘Bahia' orange juice, ^d^PCJ, pasteurized ‘Cara Cara' orange juice, ^e^total number of animals analyzed. The number in parentheses indicates the number of independent trials.

**Table 4:** Effect of pasteurized juice from ‘Bahia' and ‘Cara Cara' oranges on *C. elegans* stress resistance.

Strains and conditions	Maximum survival (hours ± SEM)	% maximum survival time variation vs. untreated	Mean survival (hours ± SEM)	% mean survival time variation vs. untreated	*p* value (log rank) POJ^a^ vs. untreated^b^	*p* value (log rank) PBJ^c^ vs. PCJ^b,d^	*N* ^e^
WT on *E. coli* OP50							
Untreated	20.33 ± 1.15		16.50 ± 0.16				145 (3)
2% Bahia	32.00 ± 1.73	57.0	27.72 ± 0.47	68.0	<0.0001		144 (3)
2% Cara Cara	35.00 ± 1.73	71.0	28.63 ± 0.47	73.5	<0.0001	0.0134	149 (3)
WT on *E. coli* OP50-HK							
Untreated	20.00 ± 1.00		15.44 ± 0.28				126 (3)
2% Bahia	23.66 ± 0.58	14.0	17.14 ± 0.33	11.0	<0.0001		134 (3)
2% Cara Cara	26.66 ± 0.58	28.0	20.42 ± 0.26	32.0	<0.0001	<0.0001	131 (3)
*skn-1(zu67)* on *E. coli* OP50							
Control	11.33 ± 0.58		9.08 ± 0.10				150 (3)
2% Bahia	11.00 ± 1.00	0.0	8.90 ± 0.11	-2.0	0.2405		150 (3)
2% Cara Cara	11.67 ± 0.58	0.0	9.14 ± 0.09	1.0	0.7441	0.1396	150 (3)
*daf-16(mu86)* on *E. coli* OP50							
Control	12.00 ± 1.73		5.84 ± 0.15				146 (3)
2% Bahia	15.00 ± 1.73	21.0	7.26 ± 0.26	24.0	<0.0001		147 (3)
2% Cara Cara	18.00 ± 1.73	42.0	8.30 ± 0.35	42.0	<0.0001	0.0097	148 (3)
*hsf-*1*(sy441)* on *E. coli* OP50							
Control	18.00 ± 1.73		14.67 ± 0.22				141 (3)
2% Bahia	23.00 ± 0.00	15.0	17.60 ± 0.38	20.0	<0.0001		150 (3)
2% Cara Cara	25.00 ± 1.73	30.0	19.86 ± 0.37	35.0	<0.0001	<0.0001	150 (3)

HK: heat killed. POJ, pasteurized orange juice, ^b^comparisons were performed using log-rank (Mantel-Cox) test. ^c^PBJ, pasteurized ‘Bahia' orange juice, ^d^PCJ, pasteurized ‘Cara Cara' orange juice, ^e^Total number of animals analyzed. The number in parentheses indicates the number of independent trials.

**Table 5:** Effect of pasteurized juice from ‘Bahia' and ‘Cara Cara' oranges on *C. elegans* paralysis induced by *β*-amyloid expression.

Strain and conditions	Mean paralysis time (hours ± SEM)	% mean paralysis time variation vs. control	*p* value (log rank) POJ^a^ vs. untreated^b^	*p* value (log rank) PBJ^c^ vs. PCJ^b,d^	*N* ^e^
CL2006 on *E. coli* OP50					
Untreated	4.59 ± 0.11				84 (3)
2% Bahia	5.75 ± 0.12	25.0	<0.0001		85 (3)
2% Cara Cara	6.70 ± 0.13	46.0	<0.0001	<0.0001	84 (3)
CL2006 on *E. coli* OP50-HK^a^					
Untreated	5.29 ± 0.16				86 (3)
2% Bahia	6.50 ± 0.18	23.0	<0.0001		80 (3)
2% Cara Cara	7.82 ± 0.22	48.0	<0.0001	<0.0001	82 (3)
CL2006 on *control(RNAi)*					
Untreated	4.80 ± 0.10				88 (3)
2% Bahia	5.48 ± 0.12	14.0	<0.0001		89 (3)
2% Cara Cara	5.54 ± 0.16	15.0	<0.0001	0.5562	89 (3)
CL2006 on *skn-1(RNAi)*					
Untreated	4.20 ± 0.07				87 (3)
2% Bahia	3.95 ± 0.07	-6.0	0.0191		88 (3)
2% Cara Cara	3.94 ± 0.07	-6.1	0.0151	0.8555	89 (3)
CL2006 on *daf-16(RNAi)*					
Untreated	4.09 ± 0.07				89 (3)
2% Bahia	4.13 ± 0.08	0.9	0.6016		88 (3)
2% Cara Cara	4.17 ± 0.08	1.9	0.4024	0.8582	89 (3)
CL2006 on *hsf*-*1(RNAi)*					
Untreated	4.26 ± 0.08				88 (3)
2% Bahia	4.57 ± 0.08	7.2	0.0069		89 (3)
2% Cara Cara	3.84 ± 0.07	-9.8	<0.0001	<0.0001	89 (3)

HK: heat killed, ^a^POJ, pasteurized orange juice, ^b^comparisons were performed using log-rank (Mantel-Cox) test. ^c^PBJ, pasteurized ‘Bahia' orange juice, ^d^PCJ, pasteurized ‘Cara Cara' orange juice, ^e^total number of animals analyzed. The number in parentheses indicates the number of independent trials.

## Data Availability

All data used to support the findings of this study are available from the corresponding author upon request.
